# Evaluation of an Innovative Colon Capsule Endoscopy Service in Scotland From the Perspective of Patients: Mixed Methods Study

**DOI:** 10.2196/45181

**Published:** 2023-04-14

**Authors:** Sarah Bond, Charalampos Kyfonidis, Matthew Jamieson, Roma Maguire, Lisa McCann, Angus Watson, Michelle Brogan, Marilyn Lennon

**Affiliations:** 1 School of Nursing and Paramedic Science Ulster University Londonderry United Kingdom; 2 Centre for Research and Technology Hellas Thessaloniki Greece; 3 School of Health & Wellbeing University of Glasgow Glasgow United Kingdom; 4 Department of Computer and Information Sciences University of Strathclyde Glasgow United Kingdom; 5 NHS Highland Inverness United Kingdom; 6 Digital Health & Care Innovation Centre Glasgow United Kingdom

**Keywords:** digital health, patient experience, colonoscopy, colon capsule endoscopy

## Abstract

**Background:**

Colonoscopy is the gold standard for lower gastrointestinal diagnostics. The procedure is invasive, and its demand is high, resulting in long waiting times. Colon capsule endoscopy (CCE) is a procedure that uses a video capsule to investigate the colon, meaning that it can be carried out in a person’s own home. This type of “hospital-at-home” service could potentially reduce costs and waiting times, and increase patient satisfaction. Little is currently understood, however, about how CCE is actually experienced and accepted by patients.

**Objective:**

The aim of this study was to capture and report patient experiences of the CCE technology (the capsule and associated belt and recorder) and of the new clinical pathway for the CCE service being rolled out as part of routine service in Scotland.

**Methods:**

This was a mixed methods service evaluation of patient experiences of a real-world, deployed, managed service for CCE in Scotland. Two hundred and nine patients provided feedback via a survey about their experiences of the CCE service. Eighteen of these patients took part in a further telephone interview to capture more in-depth lived experiences to understand the barriers and opportunities for the further adoption and scaling up of the CCE service in a way that supports the patient experience and journey.

**Results:**

Patients overall perceived the CCE service to be of significant value (eg, mentioning reduced travel times, reduced waiting times, and freedom to complete the procedure at home as perceived benefits). Our findings also highlighted the importance of clear and accessible information (eg, what to expect and how to undertake the bowel preparation) and the need for managing expectations of patients (eg, being clear about when results will be received and what happens if a further colonoscopy is required).

**Conclusions:**

The findings led to recommendations for future implementations of managed CCE services in National Health Service (NHS) Scotland that could also apply more widely (United Kingdom and beyond) and at a greater scale (with more patients in more contexts). These include promoting CCE with, for, and among clinical teams to ensure adoption and success; capturing and understanding reasons why patients do and do not opt for CCE; providing clear information in a variety of appropriate ways to patients (eg, around the importance of bowel preparation instructions); improving the bowel preparation (this is not specific to CCE alone); providing flexible options for issuing and returning the kit (eg, dropping off at a pharmacy); and embedding formative evaluation within the service itself (eg, capturing patient-reported experiences via surveys in the information pack when the equipment is returned).

## Introduction

Colon cancer is the fourth most common cancer worldwide [1]. Optical colonoscopy (OC) for colorectal screening and investigation is considered the gold standard and predominant investigative procedure in cases of suspected colorectal cancer [2]. Although prognosis with OC can improve with early detection [2], patient feedback indicates that this procedure is often uncomfortable for patients within a hospital setting [3,4]. In addition to this, there is a high demand for OC, and the available resources (staff and clinics) often cannot cover this demand, resulting in long waiting lists [5]. At the end of September 2022, 14,477 patients were waiting for a colonoscopy, with 8541 patients waiting longer than the recommended 6 weeks to access diagnostics [6]. The discomfort for patients and the burden on health services to provide a high number of OCs indicate that it is a priority to develop innovative solutions that can reduce the number of colonoscopies that are carried out [5], moving away from reliance on hospital-based diagnostics and promoting hospital-at-home solutions where appropriate.

**Figure 1:**
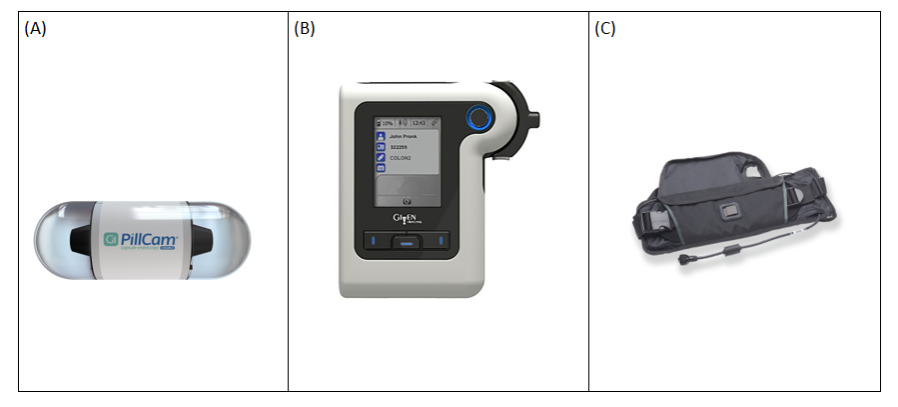
The Medtronic PillCam device used in the Scotland Colon Capsule implementation. (A) PillCam device (size, 32.3×11.6 mm); (B) Reader; (C) Belt.

New technology-enabled procedures have emerged in recent years, including colon capsule endoscopy (CCE). CCE involves a small video capsule (the size of a large vitamin pill [32.3×11.6 mm]; Figure 1A), which can be swallowed [7]. The capsule travels through the bowel and takes up to 35 images per second, which are transferred to a digital recorder (Figure 1B) worn on a belt (Figure 1C). The use of CCE within clinical practice may reduce waiting times for patients and poses a promising alternative to colonoscopy for some patients, as it does not require specialized medical facilities, meaning it can be carried out closer to patients’ homes [5]. Companies with appropriate approvals, training, and staff could provide new managed colonoscopy services where patients (where appropriate) can receive the new CCE device instead of following the existing colonoscopy route. This type of new managed service model could greatly reduce the burden on health services [5] carrying out OCs and improve patients’ experiences of the colonoscopy diagnostic procedure.

Clinical trials have already shown that CCE is accurate and safe for use, and has many potential benefits for adoption in the health care setting [8]. In terms of implementing the CCE service as a standard practice, a comprehensive report has been produced by the European Society of Gastrointestinal Endoscopy (ESGE) [9]. The ESGE presented recommendations about the use of CCE, but the report did not mention other critical aspects for adoption, such as patient experience/acceptance or barriers and facilitators to implementing the service in practice. Although innovative technologies, such as CCE, may “work,” evaluations should be expanded to answer the question, “does this work in practice?” [10]. It is also crucial to understand who might benefit the most from the technology and service, and in what contexts (which patients and which service models). Understanding the experiences of the primary users or consumers of both the technology and the service can play a key role in the establishment of a more successful new pathway and the likelihood that it is adopted and scaled [11-14].

We present the results of a large-scale national evaluation in Scotland where CCE was introduced as an alternative pathway to colonoscopy for defined patient groups (the clinical element of the study evaluation has been presented by MacLeod et al [15]). The focus of this paper is on patient acceptance and experience of CCE and the new care pathway introduced.

## Methods

### Study Design

This study was a mixed methods process service evaluation (survey and interview) from the patient experience perspective. There was no control group as the new service was being offered to all patients as part of routine care in the 3 health boards where it was being made available at the time of the study. The aim was to capture user (patient) experiences and to use these to directly inform the future design of both the technology and the service delivery model in Scotland and to provide recommendations for improved patient experience in the new CCE pathway in Scotland and beyond. 

### Ethics Approval

This study involved a service evaluation. Approval was granted by the Department of Computer and Information Science Ethics Committee, University of Strathclyde on February 8, 2019 (ID947). 

### Recruitment

Patients receiving the new CCE procedure (June-December 2019) were from 3 health boards in Scotland, United Kingdom, who had agreed to offer the service to evaluate its viability in practice. Participants were patients from the following two categories: (1) patients presenting to their general practitioner (GP) with gastrointestinal symptoms suspected to be caused by colon cancer (symptomatic patients) and (2) patients who had symptoms in the past and are under surveillance (surveillance patients). Patients were offered the CCE option as part of routine care delivery. The inclusion criteria were as follows: (1) age of 18 years or over, (2) ability to speak and read English, (3) ability to provide consent for the CCE procedure, and (4) identification as a symptomatic patient (with or without fecal immunochemistry test [FIT], full blood count [FBC], and serum biochemistry test results available, and who has been referred by a GP to the hospital for further assessment of lower gastrointestinal symptoms) or a surveillance patient scheduled for a colonoscopy. The exclusion criteria were as follows: (1) medically unfit to undergo full bowel preparation, (2) difficulty in swallowing tablets, (3) constipation or colonic slow transit as a predominant symptom (symptomatic patients), (4) microcytic anemia or diarrhea as a predominant symptom, (5) indwelling pacemaker, and (6) FIT values >400 μg/g.

All patients who opted for CCE (733 patients offered; 509 accepted [316 symptomatic patients and 193 surveillance patients]) were given an evaluation survey (Multimedia Appendix 1) after the procedure and were invited to opt into a follow-up service evaluation interview to capture their lived experiences of the CCE procedure. A total of 18 interview participants were selected using opportunistic sampling from those who responded to the opt in to interview request from National Health Service (NHS) partners and not directly from the researchers of this study.

The researcher SB contacted each person by telephone, who opted in for an interview (October-December 2019), until we reached 18 participants. Our original target was 30 participants, but due to significant delays in the service being implemented, we had to reduce this number to complete the study before the funding period ended. 

### Overview of the New CCE Service

If CCE was deemed appropriate for a patient by a clinician, the patient received bowel cleansing pharmaceuticals at home and was informed about the procedure and bowel cleansing regime. On the day of the CCE, the patient travelled to a local community health center for investigations. A nurse at the center provided the patient with information and support in swallowing the pill and wearing the recorder belt, and informed the patient when to take the boosters. The patient left the center with the belt after having swallowed the capsule and then travelled to their home. See Multimedia Appendix 2 for the full patient journey and clinical pathway for the CCE pathway.

Patients were instructed to take further bowel preparation pharmaceuticals (boosters) at regular intervals during the daytime to increase the speed of the pill’s transition through the bowel, as the camera has a battery life of around 12 hours. Pictures of the colon taken by the camera in the capsule are transmitted wirelessly from the pill to a worn belt (Figure 1C). During an investigation, the camera takes more than 50,000 images of the intestinal lining. These are then sent wirelessly to a data recorder worn around the waist and over the shoulder (Figure 1B).

The capsule takes between 6 and 12 hours on average to pass through a patient’s system, and only about 50% of patients know they have excreted the capsule. From the reporting software, we do however know when a patient excretes the capsule and how long it has taken them to excrete it (if they have). When the capsule was excreted, the patient returned the belt and recorder (Figure 1B and 1C) to the center or to their GP the next day. The results were then retrieved from the belt via a USB docking station by a nurse, and the data were transmitted electronically to a clinician for assessment. This involved watching the full video and noting the pathology observed (eg, polyps or cancerous growths). A report was then compiled and sent to the referring clinician. The results from the capsule endoscopy were then reviewed by a consultant who made a diagnosis. The diagnosis and any follow-up care were then reported back to the patient (usually within 5-10 working days), and the patient was informed if they needed any further treatment or appointments. Currently, 35% of patients need a colonoscopy and 23% need a flexible sigmoidoscopy after CCE. Patients are told that a proportion of patients will need follow-up endoscopy if any pathology is found or the test has failed.

### Data Collection

A paper-based survey was distributed to all 317 patients who received the CCE service during the evaluation period. The survey included 4 main questions capturing the reason for participation, communication about the procedure, CCE experience, and OC experience. Surveys were distributed and collected by the service provider company on behalf of the evaluators as part of routine care delivery. Follow-up semistructured interviews (Multimedia Appendix 3) were conducted by the researcher SB with 18 patients who opted in via NHS partners providing the CCE service. This took place as soon as the patient contact details (of those who opted in) were sent to our research team (SB) by the health board providing the service (typically a month after receiving the survey). Interviews were conducted (by SB) via telephone, and participants provided verbal consent to a consent form that was read to them over the phone. Patients were sent a consent form by post to read in advance. The researcher SB is a registered nurse and is an experienced researcher with a PhD involving mixed methods. SB was not known to the participants prior to the study. 

### Data Analysis

Survey and interview data were transcribed and uploaded into a qualitative data management and analysis software package (NVivo 8.0) [16]. The quantitative data were analyzed by the researcher CK using Microsoft Excel (Microsoft Corp). A thematic coding strategy was used where the nonadoption, abandonment, scale-up, spread, and sustainability (NASSS) framework’s constructs [10] were used as a priori themes for organizing and coding the data. This framework allows an understanding of the data according to the following key factors or domains: condition or illness, technology, value proposition (perceived value of service), adopter system (comprising professional staff, patients, and lay caregivers), organization, wider (institutional and societal) context, and interaction and mutual adaptation between all these domains over time. One researcher (SB) read the interview transcripts, and 4 (25%) of these were read by ML to agree on the coding process. The 2 researchers (SB and ML) agreed on how excerpts of quotes provided by each patient and across the patient group could be coded to the NASSS constructs. A third researcher (RM) reviewed the themes and exemplar quotes before the final coding and the themes were agreed. Results were then grouped and have been presented in detail in this paper with illustrative quotes where appropriate.  

## Results

### Respondents and Response Rate

Among the 317 patients who received the new CCE procedure (during the evaluation timeline of the study period), 211 (66.6%) completed a survey and 18 (5.7%) were also interviewed. From among the 211 survey respondents, we had demographic data for 183 respondents (the data of the remaining patients were not transferred during the study period) (Table 1). There were slightly more responses from males than females. Moreover, there were slightly more respondents among those who were symptomatic (n=101) than among those in the surveillance group (n=82). The average age of the respondents was 64.8 years.

**Table 1:** Overview of the demographic details of the participants who completed the surveys.

Characteristic		Value
**Sex (N=183), n**		
	Male	93
	Female	90
**Group (N=183), n**		
	Surveillance	82
	Symptomatic	101
**Reason for surveillance (N=82), n**		
	Previous polyps	49
	Colorectal cancer follow-up	23
	Hereditary nonpolyposis colorectal cancer	5
	Family history	5
**Age findings**		
	Average age (years)	64.8
	Oldest age (years)	83
	Youngest age (years)	34

Our summary of the findings presented in this paper includes responses from both the completed surveys and the follow-up interviews to understand patient experiences. The findings were thematically organized and mapped to only 2 primary domains of the NASSS framework: technology and value proposition (Table 2). The findings were also used to create a patient journey process map (Multimedia Appendix 2). This diagram also summarizes the opportunities for improvement along the CCE pathway.

**Table 2:** Key themes from patient experience (surveys and interviews).

NASSS^a^ domain	Study subthemes identified
1. Technology	1.1. Bowel cleansing 1.2. Colon capsule endoscopy capsule 1.3. Belt and holster 1.4. Results
2. Value proposition	2.1. A managed service model 2.2. Information & communication 2.3. Impact on daily life 2.4. Comparing colon capsule endoscopy to optical colonoscopy

^a^NASSS: nonadoption, abandonment, scale-up, spread, and sustainability.

### Theme 1: Technology

#### Subtheme 1.1: Bowel Cleansing

Bowel preparation was the most commented on aspect from the surveys (n=52). Most comments were negative (n=48), some were neutral (n=4), and only one was positive. A small number of patients (n=3) reported that the bowel preparation was ineffective in cleaning the bowel.

Negative impressions about the bowel preparation (including cleanser and booster) were reported as being due to (1) the substantial amount of liquid and the length of the process (which is a larger amount and longer process than routine endoscopy) (n=28), (2) bad taste (n=13), and (3) pain or discomfort due to the preparation (n=8).

Well, if they could improve the texture so that it’s more like water and not so syrupy. The nurse did say you can use a straw, but I don’t think that would make any difference, I think it would just prolong the agony really, taking it.

Patient interview #124

Negative experiences reported also included taking longer than previous experiences of laxatives, feeling unwell, feeling bloated, taking longer than expected for normal bowel motions, pain or discomfort, bleeding, toileting accidents, and requiring the support of relatives in daily tasks, such as showering and driving to appointments. It is important to note however that bowel preparation is not unique to the CCE procedure, and many of these issues would occur when bowel preparation is undertaken for routine OC as well.

Suggestions to improve the process include improving the taste, improving the quantity of the fluid, changing the texture, drinking the mixture cold, drinking the mixture concentrated followed by plain water, using a straw, using a barrier cream (a cream to put around the anus to prevent soreness) before taking the laxatives, and suggesting laxatives according to weight.

#### Subtheme 1.2: CCE Capsule 

Some patients (n=19) reported that the capsule was easy to swallow irrespective of its fairly large size (which worried them initially). Dissatisfaction after swallowing the capsule was however noted by some patients (n=17) who completed the survey and were interviewed. Half of those who voiced this indicated pain and discomfort (n=10), some noted the prolonged fasting period (n=9), and others indicated insecurity due to the lack of medical staff around them (n=3). Some patients could not determine whether the capsule had been excreted (n=4). Three patients mentioned pain and discomfort during the excretion of the capsule.

#### Subtheme 1.3: Belt and Holster 

The belt and holster were the second most mentioned aspect of the CCE service from the survey responses. Reasons for negative comments about the belt and holster were related to its current design (size, weight, and fit). Some survey patients reported that the belt and recorder were heavy and bulky (n=15), uncomfortable and cumbersome to wear all day (n=14), not steady and requiring constant adjustment (n=6), and restricting activities (n=6). The signaling mechanism of the belt and holster was reported as not being clearly understood by some patients (n=7). Similar feedback, captured in patient interviews, detailed that the equipment could not be easily hidden, which might be problematic for some individuals.

I wouldn’t want to be sitting in an office or something like that with all that on, it would be extremely obvious that you’re doing something like that.

Patient interview #83

This suggests that the comfort and wearability of the equipment are important to continuously improve upon and that there may be social stigma factors that affect perception when wearing the equipment in public settings.

#### Subtheme 1.4: Results 

Patient perspectives were captured at varying time points in the different data collection activities. Surveys mostly captured the perspectives of patients immediately following the investigation (prior to results). Interviews captured the perspectives of patients weeks to months after the CCE (after patients received the results of the CCE procedure). Two patients described a positive experience with the results, even though a pathological finding was noted. Three patients expressed a neutral tone, referring to the time the results took to be issued. Three patients reported a negative experience either because they had to wait for the results and then had to wait for a colonoscopy or because the bowel preparation was not successful and thus the results were not clear.

I know the letter’s very reassuring to say that there’s nothing serious, but at the same time if I had gone and got the old-style check possible those polyps would have been removed by now.

Patient interview #34

Future evaluations should follow-up with patient cohorts far enough along the patient journey to capture the experiences of those having different pathologies, or undergoing treatments or follow-up colonoscopies in order to better tease out the true perceived benefits of CCE versus traditional colonoscopy.

### Theme 2: Value Proposition (New Managed CCE Service)

An important aspect of this evaluation was to understand what patients’ expectations and experiences were of the managed service model for delivering CCE, as well as the capsule and the procedure itself. Subthemes were generated based on the value proposition criteria of the NASSS framework, and all were related to information, communication, and support around the service that led to perceived value (or lack of value) of the service as a whole.

#### Subtheme 2.1: Managed Service Model 

A total of 211 patients were asked about their views of this service being outsourced from the NHS to a private company within the survey, and 174 patients responded to this item. Most patients (n=150) responded that “it didn’t bother me at all,” and the rest responded either “it bothered me a little” (n=22) or “it bothered me a lot” (n=2).

Some patients further noted that the company had expertise and dealt professionally with them (n=29), and others noted it was acceptable if the result is good and improves health care, without adding extra cost (n=34).

Does it really matter who provides the service as long as it advanced healthcare.

Patient survey #50

There were however few concerns about privatization of the NHS (n=5), and some patients stated that they trusted the judgement of the NHS before private companies (n=4).

#### Subtheme 2.2: Information and Communication 

Most feedback related to the quality and importance of the provided information and communication was positive (111/151). Patients perceived the staff to be helpful (n=14), friendly (n=74), pleasant (n=11), and reassuring (n=7), and commented that they found the telephone calls highly informative (n=42). However, some patients (n=46) were dissatisfied with the information provided because they perceived the information to be inadequate (n=27) or not clear (n=26). Some patients reported that they felt uninformed after swallowing the capsule (n=9) and about the process involved after the procedure (n=5). Others found the amount of information provided to be overwhelming (n=4). Most patients expressed some misunderstanding about the level of involvement needed for the procedure (n=15). Some patients (n=11) expressed confusion with the entire process, including the preparation (n=5). Lack of information about the risks of CCE and its advantages and disadvantages were also reported (n=3).

#### Subtheme 2.3: Impact on Daily Life and Routine

Survey feedback indicated that some patients (n=10) felt the CCE procedure had a negative impact, but many (n=72) also stated that it had a positive impact on their daily life and routine. Positive feedback related to the procedure involved less travel (n=10) and the ability to complete most aspects at home (n=17). This allowed the patients flexibility to do other things (n=14), saving time (n=26) and allowing continuation of their normal routine.

During interviews, however, many participants described how their daily activities were limited or negatively impacted while taking the laxatives and capsule, and wearing the recording belt during the imaging period. Activities that were altered significantly included not working or showering, and staying indoors with washroom facilities nearby. This is illustrated by the following participant quote:

Four days I would have- I had the Monday off, there was absolutely no way, I spent most of Tuesday in my bed I was so weak and if I was out of my bed I was just sitting on the sofa. Wednesday I wasn’t well enough to go to work…Yeah … it would have been four days off work….

Patient interview #113

Throughout interviews, some patients (n=11) mentioned negative aspects about travelling due to the challenge of travelling with bowel preparation (n=6) and the distance they had to travel (n=4). Other patients (n=8), however, commented on how it made travel less of a burden for them. This is illustrated in the following participant quote:

Well I’d say it impacted it positively because I was able to spend most of the day working from home. Which is not something that you’d be able to do if you had to go into hospital for a colonoscopy, so it’s a positive impact.

Patient interview #30

#### Subtheme 2.4: Comparing CCE to OC

Reasons for preferring CCE to OC included the perception that it was less invasive and embarrassing (n=40), easier (n=20), less painful (n=26), quicker (n=13), less stressful (n=8), more effective (n=7), and better for the NHS (n=4). Reasons for preferring colonoscopy included bowel preparation (n=7), pain and discomfort (n=5), efficiency (time needed and ability to find and treat a pathology) (n=16), possibility of still needing further investigations if undergoing CCE (n=8), and others (pain and discomfort, fasting, problems during the procedure, information given, and use of a belt).

The down sides were for me the delay in getting the results. If I’d had a traditional colonoscopy then when they saw the polyps, they could have removed them then and done the biopsy, and everything would be out of the way in one go.

Patient interview #83

Only 1 patient stated that they would not recommend CCE to others as they thought the process was too lengthy. The majority (n=162) of patients responded positively, indicating they would suggest CCE to others. Six patients responded negatively, and 29 were not sure if they would recommend CCE. Ninety patients provided further explanation, with positive and negative personal experiences as reasons (n=56).

I'd go for CCE way before any 'scope'

Patient survey #128

Sixteen of those patients who were unsure whether to recommend this investigation to others provided further explanation. The reasons included feeling unsure of the effectiveness of CCE (n=4), difficulty with fasting and bowel preparation (n=4), possibility of still needing colonoscopy (n=2), and a desire to not influence others (n=1).

## Discussion

This evaluation revealed that patients were accepting of a new managed service for CCE in 3 health boards in Scotland, United Kingdom. The benefits included being able to stay at home, reduced reliance on family, and less need to travel. Our findings revealed that managed service models for technology-enabled care pathways can be perceived as acceptable to patients. One limitation of this study was that we were unable to follow-up with patients at the phase where they would have received their results or would have gone for a follow-up colonoscopy or started treatment. This was due to the fact that this evaluation was being conducted as a service evaluation in order to inform further implementations of the service, which were being planned already at the time of this data collection. This limited the timeframe for the data collection. This follow-up could provide more insights into different patient experiences depending on what happens after the CCE procedure, and as a result of this study, this is now being included in future evaluations of CCE in Scotland.

Our findings revealed negative experiences with the bowel preparation for CCE; however, these are to be expected as the bowel preparation in regular colonoscopy screening is also cited as being unpleasant or involving toleration difficulty, and thus, our findings are in alignment with previous literature [17-20]. Based on patient feedback, the bowel preparation regime should continue to be developed in the future to improve the volume and taste of the cleansers being used. Providing more information, however, on the bowel preparation for both alternative procedures might be a good improvement from the patient experience perspective to manage expectations [20]. The importance of complying with the bowel preparation regimen should also be emphasized, as this maximizes the chances of a successful test [21-23].

Several improvements to the hardware or accompanying kit (the belt and recorder device) were suggested by the patients (in terms of its form factor and comfort). The belt and holster have already undergone design refinements based upon this evaluation’s preliminary findings. Innovative technologies for health and care need to be designed to be usable, wearable, comfortable, and acceptable to patients. Capturing the experiences of such kits, including the social stigma associated with using or wearing them, needs to be an ongoing evaluation activity to improve the overall patient experience and increase the likelihood of acceptance and adoption more widely.

The information patients received was of great importance to them. Clear information (eg, verbal and written) should be given to patients so that they can know what to expect prior to the procedure and know their role within the procedure (eg, boosters being laxatives, risks associated with CCE, size and weight of the belt, results, length of the procedure, amount of bowel preparation, comparison with standard colonoscopy, and how and when patients will receive the results). Staff should also clearly point out that a follow-up colonoscopy in some cases is still required and explain why this is the case (and how likely). This information needs to be accessible to a wide range of patients with different expectations and diverse levels of health and digital literacy.

A variety of patient experience measures, such as those collected in this evaluation, should be collected during and throughout future scaling up and roll out of CCE services, rather than continuing to rely on separate add-on studies for capturing patient acceptance. In this way, new products and services can be improved on a continuous basis and will result in better experiences more quickly for patients.

## Appendix

Multimedia Appendix 1 Patient survey, including the consent process.

Multimedia Appendix 2 Key findings mapped to the patient pathway.

Multimedia Appendix 3 Patient interview: information sheet and topic guide.

## Abbreviations

CCE: colon capsule endoscopy
ESGE: European Society of Gastrointestinal Endoscopy
FIT: fecal immunochemistry test
GP: general practitioner
NASSS: nonadoption, abandonment, scale-up, spread, and sustainability
NHS: National Health Service
OC: optical colonoscopy
