# Supplementary material for: Evaluation of an Innovative Colon Capsule Endoscopy Service in Scotland From the Perspective of Patients: Mixed Methods Study
Source: J Med Internet Res. 2023 Apr 14;25:e45181. doi: 10.2196/45181 (PMC10148218; doi:10.2196/45181)
Supplement: Multimedia Appendix 1 [file jmir_v25i1e45181_app1.docx]

# Multimedia Appendix 1. Patient survey, including the consent process.

## 1) Initial contact details consent form

|  |
| --- |

## 2) ScotCap Patient Survey

| **ScotCap: Patient Survey**  **A service evaluation of colon capsule endoscopy in patients with gastro-intestinal symptoms**  Please complete this survey immediately after using the Colon Capsule Endoscopy (CCE).  After completion, please pass back to the Corporate Health International (CHI) team at the same time as you are returning your belt and recording equipment for the CCE procedure.  **Please do not include any personal details (for example your name or address) in your responses on this survey.**  **Question 1**   \| **Why did you decide to take the CCE rather than have a colonoscopy? (tick all those that apply)** \| \| \| --- \| --- \| \| I wanted to help improve healthcare \|  \| \| I wanted to help reduce healthcare costs associated with colonoscopy \|  \| \| I wanted to avoid a colonoscopy: \|  \| \| **What factors were important in this choice?** (tick all those that apply) \|  \| \| 1. travel \|  \| \| 1. burden to family \|  \| \| 1. time taken off work \|  \| \| 1. time in hospital \|  \| \| 1. pain as discomfort from colonoscopy procedure \|  \| \| 1. fear of undergoing a colonoscopy procedure \|  \| \| 1. ***Other: please tell us, in the space below, why you decided to use the CCE?*** \| \| \|  \| \| |
| --- | --- | --- | --- | --- | --- | --- | --- | --- | --- | --- | --- | --- | --- | --- | --- | --- | --- | --- | --- | --- | --- | --- | --- | --- | --- | --- |

| **Question 2:**  **Was your experience of taking the CCE as you expected? (please circle your response)**   \| Yes \| No \| Not Sure \| \| --- \| --- \| --- \|   **Can you explain your response?**   \|  \| \| --- \|   **To what extent did your experience of taking the CCE meet your expectations? (Please circle your response)**   \| Worse than expected \|  \|  \|  \|  \| Better than expected \| \| --- \| --- \| --- \| --- \| --- \| --- \| \| 0 \| 1 \| 2 \| 3 \| 4 \| 5 \|   **Can you explain (give examples) of when the service did or did not match your expectations?**   \|  \| \| --- \|   **Question 3:**  **Do you feel that you were fully informed about taking the CCE before you took it? (Please Circle).**   \| Yes \| No \| Not Sure \| \| --- \| --- \| --- \|   **Can you explain your response?**   \|  \| \| --- \|   **Was the information you received understandable?**   \| Yes \| No \| Not Sure \| \| --- \| --- \| --- \|   **How could this be improved?**   \|  \| \| --- \|   **Did you feel you received enough information? (Please circle your response)**   \| Yes \| No \| Not Sure \| \| --- \| --- \| --- \| |
| --- | --- | --- | --- | --- | --- | --- | --- | --- | --- | --- | --- | --- | --- | --- | --- | --- | --- | --- | --- | --- | --- | --- | --- | --- | --- | --- | --- | --- |

| **Please give examples of how the information received from healthcare professionals about the CCE could be improved?**   \|  \| \| --- \|   **Did you use the online ScotCap application (app) to find out further information? (Please circle your response)**   \| Yes \| No \| Not Sure \| \| --- \| --- \| --- \|   **If you answered yes, why did you use it?**   \|  \| \| --- \|   **If you answered yes, what improvements could be made to the online app?**   \|  \| \| --- \|   **Question 4:**  **Have you had a colonoscopy before? (Please circle your response)**   \| Yes \| No \| Not Sure \| \| --- \| --- \| --- \|   **This question is for people who have had both a previous colonoscopy and used the CCE. Based on your experience, if you needed another investigation, which would be your preference?**   \| I have no preference \| CCE \| Colonoscopy \| \| --- \| --- \| --- \|   **Why?**   \|  \| \| --- \|   **Based on your experience, would you recommend this device or service to someone else who was**  **in your position? (Please circle your answer)**   \| Yes \| No \| Not Sure \| \| --- \| --- \| --- \|   **Can you explain your response?**   \|  \| \| --- \|   **How did you feel about a company being used to provide this service with/on behalf of the NHS?**  **(Please circle your answer)**  *It bothered me a lot     It bothered me a little  It didn’t bother me at all*  **Can you explain your response?**   \|  \| \| --- \|   **Thank you for taking the time to complete this survey.**  **Please return this to Corporate Health International**  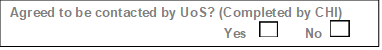 |
| --- | --- | --- | --- | --- | --- | --- | --- | --- | --- | --- | --- | --- | --- | --- | --- | --- | --- | --- |
