# Supplementary figures and images for: Evaluation of an Innovative Colon Capsule Endoscopy Service in Scotland From the Perspective of Patients: Mixed Methods Study
Source: J Med Internet Res. 2023 Apr 14;25:e45181. doi: 10.2196/45181 (PMC10148218; doi:10.2196/45181)

**Multimedia Appendix 2.** Key findings mapped to the patient pathway. *
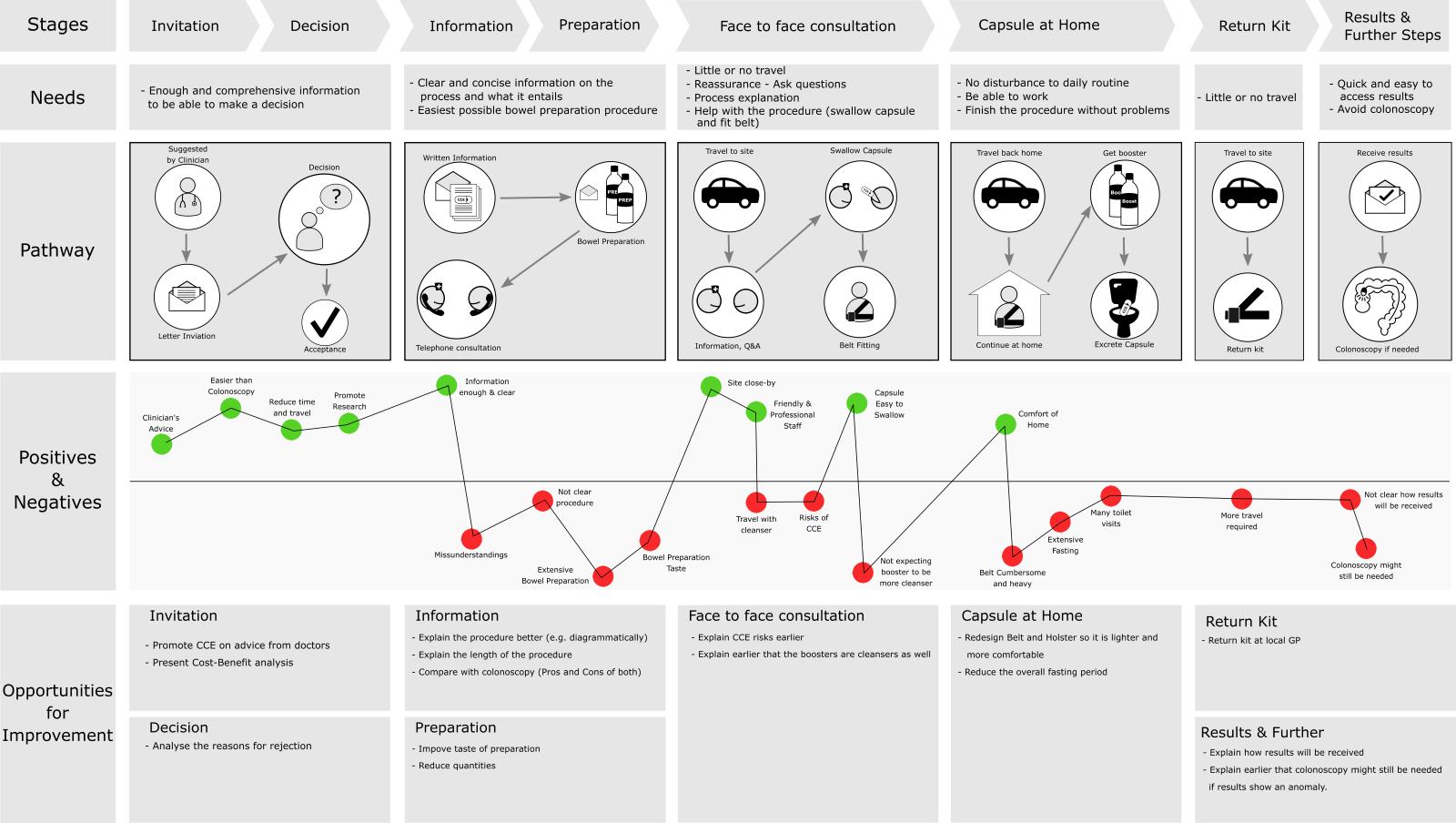
*

Supplement: Multimedia Appendix 2 [file jmir_v25i1e45181_app2.docx]
