# Supplementary material for: Evaluation of an Innovative Colon Capsule Endoscopy Service in Scotland From the Perspective of Patients: Mixed Methods Study
Source: J Med Internet Res. 2023 Apr 14;25:e45181. doi: 10.2196/45181 (PMC10148218; doi:10.2196/45181)
Supplement: Multimedia Appendix 3 [file jmir_v25i1e45181_app3.docx]

## **Multimedia Appendix 3.** Patient interview: information sheet and topic guide.

## Participant Information Sheet (Patient Interview)

| **ScotCap: Interview: Participant Information Sheet**  **A service evaluation of colon capsule endoscopy in patients with gastro-intestinal symptoms**  CHI is providing a new managed gastrointestinal screening service and the University of Strathclyde is evaluating this new service. You are invited to take part in a research project about your experience of the bowel screening services within the Highlands and Islands to help improve the service provided.  Before you decide whether to take part, it is important to understand why this research is being undertaken and what it will involve. Please read the following information and discuss with others if you wish. If you have any questions about any aspect of this project, please ask us in person or please use the contact information at the end to contact us. Please note that reading this information sheet or providing your initial contact details does not mean you are consenting to take part.  **Do you have to take part?**  No. Taking part is your decision and it is entirely voluntary.  **What is this project?**  The project involves asking people who have received gastrointestinal investigations for their views about the service they have received. By taking part in this project, you will be invited to take part in an interview about your experience with the colon capsule. The interviews would be either face to face or on the phone and will take 30 to 60 minutes. We will ask questions and we aim to understand your experiences, expectations of new and old gastrointestinal screening, and how things could be better in the future. We will audio record your responses for use in our research. This will be transcribed by professional transcribers.  **The information being collected and used**  If you consent to take part in an interview, the University of Strathclyde will contact NHS Highland (RD&I) and request your contact details using your unique pseudonym from your completed survey. RD&I will send your contact details securely to the University of Strathclyde. The University of Strathclyde will then contact you through your preferred contact method, to provide more information about the interview. If you have not consented to share your personal contact details then these will not be shared to the University of Strathclyde and the University of Strathclyde will not contact you.  If you agree to participate in the study by taking part in an interview, you will be asked to provide your consent over the phone or on a consent form. If consent is taken over the phone, this conversation will be audio recorded and transcribed. This will be used to confirm you understand your role within the project and that you are happy to participate. The conversation will start with a summary of the project and the below statements will be read and you will be asked for your consent for each statement. If you decline to provide consent this audio recording will be deleted, and the interview will not take place.  **Statements are listed below (read aloud by the researchers on phone call when taking consent):**   \| 1. *I confirm that I have read the information sheet dated 04/07/2019 (version 1) for the above*   *study. I have had the opportunity to consider the information, ask questions, and have had*  *these answered satisfactorily.* \| \| \| --- \| --- \| \| 1. *I understand that my participation is voluntary and that I am free to withdraw at any time,*   *without giving any reason, and my medical care or legal rights will not be affected.* \| \| \| 1. *I understand and agree that the interview will be audio recorded and will be typed up by a professional audio typist. The audio recordings will be securely destroyed at the end of the*   *project.* \| \| \| 1. *I understand that the data I provide will remain anonymous and only identifiable by a*   *pseudonym. I understand that anonymised data relating to this project may be looked at, or shared, with relevant individuals from the Digital Health and Care Institute, Corporate Health International (CHI) The Centre for Healthcare Randomised Trials (CHaRT) and the NHS.*  *I give permission for this.* \| \| \| 1. *I consent to my words being quoted anonymously. Quotes may be used in written journal*   *articles, reports, and thesis or at academic conferences. This may be used for the promotion*  *of the research via broadcast, digital or print media.* \| \| \| 1. *I agree to my data being used in the ways outlined in this information sheet.* \| \|   **Who is organising and funding the project?**  The University of Strathclyde, Digital Health & Care Institute (DHI), Corporate Health International (CHI) The Centre for Healthcare Randomised Trials (CHaRT) and NHS Western Isles, NHS Highland & NHS Grampian are partners on a collaborative project entitled ‘ScotCap Evaluation’ (the “Evaluation”). The Project is funded by the Digital Health & Care Institute (which is currently hosted by The University of Strathclyde).  **Who has checked this service evaluation?**  The ethics committee of Computer and Information Sciences department of University of Strathclyde has checked and approved this service evaluation study. Some of the data collected during the study may be looked at by individuals from the University of Strathclyde or from NHS Highlands and Islands to check that the service evaluation is properly conducted and the interests of those taking part are protected.  **Data controller and the data protection officer**  The University of Strathclyde is committed to transparency and to complying with its responsibilities under data protection legislation. This privacy notice sets out important information regarding how we will use your information and your rights under the legislation. It is important that you read this notice prior to providing your information. For the purposes of this evaluation, the University of Strathclyde are Data Owners of the Interviews under data protection legislation. Any enquiries regarding data protection should be made to the University’s Data Protection Officer at [dataprotection@strath.ac.uk](mailto:dataprotection@strath.ac.uk).  **Purpose of the processing and legal basis**  We will use your personal data collected in surveys and interviews only for the purposes of the ScotCap service evaluation. There is more information about the service itself in the participant information booklet you received at the start when you first decided whether to take the capsule or not. Data Protection legislation requires us to identify our legal basis for processing your personal data. As we are processing your personal data for the purposes of evaluating a service, we consider that it is necessary for the performance of a task carried out in the public interest. Some of the personal data we process will relate to your health, which is classed as “special category” personal data. We consider that the processing of this special category data is necessary for reasons of substantial public interest, specifically, it is necessary for statistical purposes, and is carried out in accordance with requirements of Data Protection legislation. At the end of the project, the partners involved in the project (University of Strathclyde, Digital Health & Care Institute, Corporate Health  International (CHI), (CHaRT) and NHS Western Isles, NHS Highland & NHS Grampian) may use the collated results (which will not include any personal data that identifies you) for further service evaluation and promotional purposes. For example, the collated results may be presented in exhibitions, publications, journal articles, conference paper/presentations, lectures or broadcasts, or be used to publicise the work of the project in exhibitions, print and broadcast media and online publication, including social media.  **Retention period or criteria used**  The University of Strathclyde will retain your personal information for ten years for administrative purposes at which point they will be deleted from the University’s files. They cannot pass these details onto anyone else outside this project team for any other purposes not described here.  **Data subject rights**  You have the right to: be informed about the collection and use of your personal data; to request access to the personal data we hold about you; you are entitled to request to have personal data rectified if it is inaccurate or incomplete; you have the right to request to object to your data being processed and you can request to restrict the processing of your personal information. To exercise these rights please contact [dataprotection@strath.ac.uk](mailto:dataprotection@strath.ac.uk). Any data that have been anonymised cannot be withdrawn once they have been included in the study.  **Right to complain to supervisory authority**  If you have any concerns/issues with the way the University has processed your personal data you can contact the Data Protection Officer at [dataprotection@strath.ac.uk](mailto:dataprotection@strath.ac.uk). You also have the right to lodge a complaint against the University regarding data protection issues with the Information Commissioner’s Office (<https://ico.org.uk/concerns/>).  **Many thanks for reading this information sheet. Should you be interested in taking part or want further information, please get in touch:**   \| **Dr Marilyn Lennon**  Reader in Digital Health and Wellbeing Group, University of Strathclyde  26 Richmond Street  Glasgow G1 1XH  Telephone: +44 (0)141 548 3098  E-mail: marilyn.lennon@strath.ac.uk \| **Sarah Horan**  Research Assistant in Digital Health and Wellbeing Group, University of Strathclyde  26 Richmond Street  Glasgow G1 1XH  Telephone: +44 (0)141 548 3138  E-mail: sarah.horan@strath.ac.uk \| \| --- \| --- \| |
| --- | --- | --- | --- | --- | --- | --- | --- | --- | --- | --- | --- | --- | --- | --- |

## Patient Interview Topic Guide

| **A service evaluation of colon capsule endoscopy in patients with gastro-intestinal symptoms**  **UoS Interview Topic Guide: Patient**  Following are the questions (prompts) we will ask as part of our qualitative (Interview) Patient experience evaluation for ScotCap.  **INTRO SCRIPT:**  **Introductions (name and role within UoS) & confirmation of suitability of time (e.g. Is now a good time to speak, or would you like me to call you back at a convenient time?)**  Thank you for agreeing to take part in this interview. As explained in the participant information sheet, a company called Corporate Health International (CHI) is providing a new managed gastrointestinal screening service and the University of Strathclyde is evaluating this new service. We would like to ask you some questions about your thoughts and experiences of this service and the capsule itself. I believe that CHI have already given you a participant information sheet, have you been able to read through this? (if yes proceed) Do you have any questions? (answer questions/if no further questions proceed). As explained in the Participant information sheet, this conversation will be recorded to document our conversation, but only to help us take notes then the audio file is deleted. If you are happy to proceed, I am going to turn on the voice recorder) *(if yes, turn on voice recorder)*  So, before we begin, I will read some statements and ask you to response with a yes or no. If there are any statements which you don’t understand, or would like me to repeat, let me know.   \| 1. *I confirm that I have read the information sheet dated 04/07/2019 (version 1) for the above study. I have had the opportunity to consider the information, ask questions, and have had these answered satisfactorily.* \| \| --- \| \| 1. *I understand that my participation is voluntary and that I am free to withdraw at any time, without giving any reason, and my medical care or legal rights will not be affected.* \| \| 1. *I understand and agree that the interview will be audio recorded and will be typed up by a professional audio typist. The audio recordings will be securely destroyed at the end of the project.* \| \| 1. *I understand that the data I provide will remain anonymous and only identifiable by a pseudonym. I understand that anonymised data relating to this project may be looked at, or shared, with relevant individuals from the Digital Health and Care Institute, Corporate Health International (CHI) The Centre for Healthcare Randomised Trials (CHaRT) and the NHS. I give permission for this.* \| \| 1. *I consent to my words being quoted anonymously. Quotes may be used in written journal articles, reports, and thesis or at academic conferences. This may be used for the promotion of the research via broadcast, digital or print media.* \| \| 1. *I agree to my data being used in the ways outlined in this information sheet.* \|   If there are any questions which you don’t understand or prefer not to answer, please let me know. If you need to have a break or wish to stop during this interview, please let me know.   1. **Can you tell us a bit about how you were introduced to the (CCE) capsule option and how you felt deciding whether to take this capsule option?**   *(Below items are to be used as prompts)*   - Who introduced the colon capsule endoscopy capsule to you? - Did they provide you with any verbal or written information? - What did you think about the information provided? - Had you heard of colon capsule endoscopy before? - How were you notified of the date of your colon capsule endoscopy? - Why did you choose the colon capsule endoscopy? How easy was it to decide to take the capsule?  1. **Can you tell us about your full experience of taking/using/having the** colon capsule endoscopy **capsule/investigation** *(prompts)*   ***Bowel Preparation***   - What did you think of the bowl preparation? - What were your experiences after taking the bowel prep? - Could anything be improved in this process?   ***The day you had your* colon capsule endoscopy*:***   - Tell us about what happened on the day you received your colon capsule endoscopy test? - Where did you go? Who saw you? - Who gave you the colon capsule endoscopy capsule/pill? - How did you find swallowing the pill? (size, weight) - Did you have any concerns about ‘passing’ the pill after the investigation? - What were your experiences of wearing the recording belt? - What happened when you returned to the clinic?   - Did you receive any information about next steps and when to expect results?   - (When) /(How) Did you receive your results after the clinic visit?   **Impact of colon capsule endoscopy on daily life:**   - How did this type of screening procedure impact your daily routine? - Did you go to work? / Undertake normal everyday activities? - How far did you have to travel to take the capsule/return the belt? - If you have received a colonoscopy in the past, do you think that the colon capsule endoscopy was more or less convenient? Did it take up more or less of your time?  1. **Do you have anything else you can you tell us overall how you feel about the experience now/so far (both positive and negative)?***(Below items used as prompts)*  - How would you feel if you were given the colon capsule endoscopy pill option again? - Would you recommend the colon capsule endoscopy to your friends and/or family?   ***THANK YOU, SCRIPT [AT END]***  Thank you very much for your time in this interview. We have finished the interview, so I am going to turn off the voice recorder now *(Turn off voice recorder).* |
| --- | --- | --- | --- | --- | --- | --- |
